# *cis*-Dichloridobis(1,10-phenanthroline)cobalt(II) dimethyl­formamide solvate

**DOI:** 10.1107/S1600536808030341

**Published:** 2008-09-27

**Authors:** Shuang-Lian Cai, Shao-Ming Ying, Hui Li, Yun Chen

**Affiliations:** a College of Chemistry and Chemical Engineering, Hunan University, Changsha, Hunan 410082, People’s Republic of China; b College of Chemistry and Chemical Engineering, Jinggangshan University, Ji’an, Jiangxi 343009, People’s Republic of China

## Abstract

In the title complex, [CoCl_2_(C_12_H_8_N_2_)_2_]·C_3_H_7_NO, which has twofold rotation symmetry, the Co^II^ cation is coordinated by two 1,10-phenanthroline (phen) mol­ecules and two chloride ligands in a distorted octa­hedral geometry. In the crystal structure, a cavity is created by six complex mol­ecules connected by C—H⋯π inter­actions and non-classical C—H⋯Cl hydrogen bonds. The cavities are occupied by the disordered dimethyl­formamide solvent mol­ecule. The C and N atoms of the C—N bond in the solvent mol­ecule also lie on a crystallographic twofold rotation axis; the remaining atoms of the solvent are statistically disordered (ratio 0.5:0.5) about this axis.

## Related literature

For general background, see: Forster *et al.* (2000 ▶); Holder *et al.* (2007 ▶); Ma *et al.* (2002 ▶). Matsumoto *et al.* (2002 ▶); Xie *et al.* (2006 ▶). For a related structure, see: Hazell *et al.* (1997 ▶).
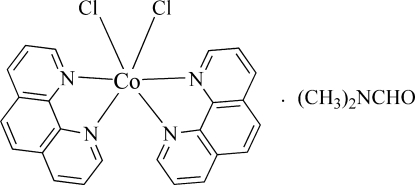


## Experimental

### 

#### Crystal data

[CoCl_2_(C_12_H_8_N_2_)_2_]·C_3_H_7_NO
*M* *_r_* = 563.33
Orthorhombic, 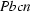

*a* = 16.345 (3) Å
*b* = 12.342 (2) Å
*c* = 12.342 (2) Å
*V* = 2489.8 (8) Å^3^
*Z* = 4
Mo *K*α radiation
μ = 0.94 mm^−1^
*T* = 293 (2) K
0.20 × 0.20 × 0.20 mm

#### Data collection

Rigaku Mercury70 CCD diffractometer
Absorption correction: multi-scan (*CrystalClear*; Rigaku & Mol­ecular Structure Corporation, 2000 ▶) *T*_min_ = 0.829, *T*_max_ = 0.829
14711 measured reflections
2204 independent reflections
2168 reflections with *I* > 2σ(*I*)
*R*_int_ = 0.017

#### Refinement

*R*[*F* ^2^ > 2σ(*F* ^2^)] = 0.026
*wR*(*F* ^2^) = 0.063
*S* = 1.09
2204 reflections
180 parameters
2 restraints
H-atom parameters constrained
Δρ_max_ = 0.34 e Å^−3^
Δρ_min_ = −0.21 e Å^−3^

### 

Data collection: *CrystalClear* (Rigaku & Molecular Structure Corporation, 2000 ▶); cell refinement: *CrystalClear*; data reduction: *CrystalClear* program(s) used to solve structure: *SHELXS97* (Sheldrick, 2008 ▶); program(s) used to refine structure: *SHELXL97* (Sheldrick, 2008 ▶); molecular graphics: *DIAMOND* (Brandenburg, 2005 ▶); software used to prepare material for publication: *SHELXTL* (Sheldrick, 2008 ▶) and *PLATON* (Spek; 2003 ▶).

## Supplementary Material

Crystal structure: contains datablocks I, global. DOI: 10.1107/S1600536808030341/sj2538sup1.cif

Structure factors: contains datablocks I. DOI: 10.1107/S1600536808030341/sj2538Isup2.hkl

Additional supplementary materials:  crystallographic information; 3D view; checkCIF report

## Figures and Tables

**Table:** 

Co1—N2	2.1517 (13)
Co1—N1	2.1636 (13)
Co1—Cl1	2.4099 (5)

**Table:** 

N2—Co1—N2^i^	176.70 (7)
N2—Co1—N1	76.81 (5)
N2—Co1—N1^i^	100.65 (5)
N1—Co1—N1^i^	82.44 (7)
N2—Co1—Cl1	91.56 (4)
N2^i^—Co1—Cl1	90.43 (4)
N1—Co1—Cl1	162.67 (4)
N1^i^—Co1—Cl1	87.23 (4)
Cl1—Co1—Cl1^i^	105.91 (2)

Symmetry code: (i) 
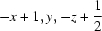
.

**Table 2:** Hydrogen-bond geometry (Å, °)

*D*—H⋯*A*	*D*—H	H⋯*A*	*D*⋯*A*	*D*—H⋯*A*
C10—H10*A*⋯Cl1	0.93	2.74	3.3408 (17)	124
C6—H6*A*⋯Cl1^ii^	0.93	2.80	3.6743 (18)	158
C5—H5*A*⋯Cl1^iii^	0.93	2.85	3.6375 (17)	144
C2—H2*A*⋯*Cg*1^iv^	0.93	2.99	3.768 (2)	142
C8—H8*A*⋯*Cg*2^v^	0.93	2.90	3.608 (2)	134

Symmetry codes: (ii) 
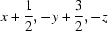
; (iii) 
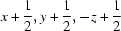
; (iv) 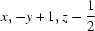; (v) 
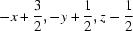
.

**Fig. 1.**
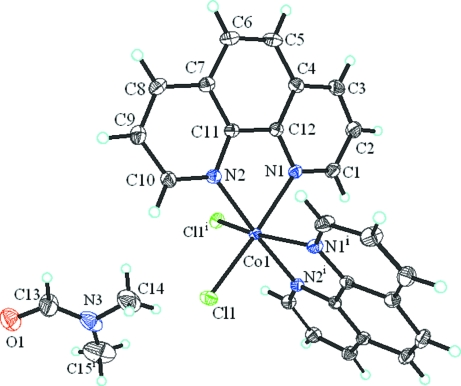
The structure of the title compound showing the atom numbering. Thermal ellipsoids are drawn at the 30% probability level and H atoms are shown as small spheres of arbitrary radii. Only one of the disorder components of the dmf molecule is shown. [Symmetry codes: (i) 1 - x + 1, y, 1/2 - z.]

**Fig. 2.**
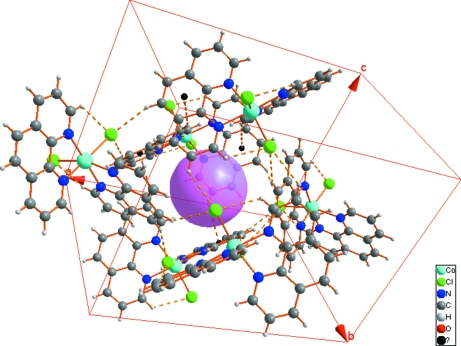
Crystal packing of (I) showing the cavity (represented by the pink sphere) created by the C—H···Cl and C—H···π interactions with hydrogen bonds drawn as dashed lines.

## supplementary crystallographic information

### Comment

*ML*_m_*X*_n_ coordination compounds (*L* = α,α'-diimine chelate ligands, such as 2,2'-bipyridine, 1,10-phenanthroline, and their derivatives; *X* = halide or pseudohalide ligands) have been receiving extensive attention due to their importance in crystal engineering and supramolecular chemistry. They also serve as models to aid the understanding of phenomena such as photosensitization and crystallization (Forster *et al.*, 2000; Holder *et al.*, 2007; Ma *et al.*, 2002). In such molecules a variety of weak intermolecular interactions involving the halide anions, aromatic ligands and solvent molecules can stabilise and regulate the supramolecular architecture in different aggregation states (Matsumoto *et al.*, 2002; Xie *et al.*, 2006). Herein, we report the crystal structure of a new cobalt(II) chloride complex with a phenanthroline ligand, Fig 1.

The crystallographic asymmetric unit of (I) consists of one half occuapncy Co^II^ atom that lies on a two-fold rotation axis, one phenanthroline molecule, one Cl^-^ anion, and half a molecule of dimethylformamide. In the complex, the Co^II^ atom is in a distorted octahedral coordination environment provided by four N atoms from two bidentate phen ligands and two terminal Cl^-^ anions. The Co—N and Co—Cl bond lengths (Table 1) are normal, and are comparable to those found in a related octahedral cobalt(II) complex [CoCl_2_(C_12_H_8_N_2_)_2_].1.5CH_3_CN [Hazell *et al.*, 1997].

Interestingly in the crystal structure, a cavity is created by six complex molecules connected by C—H···π interactions and non-classical C—H···Cl hydrogen bonds (Table 2, Fig. 2) which is occupied by the disordered dmf solvate molecule.  The solvate lies with the C14 and N3 on a crystallographic 2-fold rotation axis; the remaining atoms of the solvate are statistically disordered about this axis.  The calculated void space of the cavity was estimated to be 557.6 Å^3^ per unit cell, which corresponds to 23.2% of the total volume (2489.8 Å^3^) (Fig 2) (Spek, 2003).

### Experimental

[CoCl_2_.6(H_2_O)] (238 mg) was dissolved in a mixture of dimethylformamide (10 ml) and tetrahydrofuran (10 ml) with stirring. A color change from blue to dark blue was observed after the phenanthroline (40 mg) was added to the solution. The mixture was cooled down to room temperature after stirring for 1 h at 90 *^o^*C. The resulting mixture was then filtered, and the filtrate was concentrated to *ca* 13 ml by rotary evaporation and left in a refrigerator at 4 *^o^*C. Transparent blue prismatic crystals suitable for X-ray diffraction were produced in a few days (yield 21%). Analysis calculated for C_27_H_23_C_l2_CoN_5_O: C 57.57, N 12.43, H 4.12%; found: C 57.72, N 12.56, H 3.97%.

### Refinement

The H atoms bonded to C atoms were placed in calculated positions and treated using a riding-model approximation (C—H = 0.96 Å and *U*_iso_(H) = 1.5*U*_eq_(C) for the methyl group; C—H = 0.93 Å and *U*_iso_(H) = 1.2*U*_eq_(C) for the 1,10-phenanthroline and aldehyde groups).

### Crystal data

**Table:**

[CoCl_2_(C_12_H_8_N_2_)_2_]·C_3_H_7_NO	*F*(000) = 1156
*M**_r_* = 563.33	*D*_x_ = 1.503 Mg m^−^^3^
Orthorhombic, *P**b**c**n*	Mo *K*α radiation, λ = 0.71073 Å
Hall symbol: -P 2n 2ab	Cell parameters from 6370 reflections
*a* = 16.345 (3) Å	θ = 2.1–25.0°
*b* = 12.342 (2) Å	µ = 0.94 mm^−^^1^
*c* = 12.342 (2) Å	*T* = 293 K
*V* = 2489.8 (8) Å^3^	Block, colorless
*Z* = 4	0.20 × 0.20 × 0.20 mm

### Data collection

**Table:**

Rigaku Mercury70 CCD diffractometer	2204 independent reflections
Radiation source: fine-focus sealed tube	2168 reflections with *I* > 2σ(*I*)
graphite	*R*_int_ = 0.017
ω scans	θ_max_ = 25.0°, θ_min_ = 2.1°
Absorption correction: multi-scan (CrystalClear; Rigaku & Molecular Structure Corporation, 2000)	*h* = −17→19
*T*_min_ = 0.829, *T*_max_ = 0.829	*k* = −14→14
14711 measured reflections	*l* = −13→14

### Refinement

**Table:**

Refinement on *F*^2^	Primary atom site location: structure-invariant direct methods
Least-squares matrix: full	Secondary atom site location: difference Fourier map
*R*[*F*^2^ > 2σ(*F*^2^)] = 0.026	Hydrogen site location: inferred from neighbouring sites
*wR*(*F*^2^) = 0.063	H-atom parameters constrained
*S* = 1.09	*w* = 1/[σ^2^(*F*_o_^2^) + (0.0288*P*)^2^ + 1.9948*P*] where *P* = (*F*_o_^2^ + 2*F*_c_^2^)/3
2204 reflections	(Δ/σ)_max_ < 0.001
180 parameters	Δρ_max_ = 0.34 e Å^−^^3^
2 restraints	Δρ_min_ = −0.21 e Å^−^^3^

### Special details

**Table:**

Geometry. All e.s.d.'s (except the e.s.d. in the dihedral angle between two l.s. planes) are estimated using the full covariance matrix. The cell e.s.d.'s are taken into account individually in the estimation of e.s.d.'s in distances, angles and torsion angles; correlations between e.s.d.'s in cell parameters are only used when they are defined by crystal symmetry. An approximate (isotropic) treatment of cell e.s.d.'s is used for estimating e.s.d.'s involving l.s. planes.
Refinement. Refinement of *F*^2^ against ALL reflections. The weighted *R*-factor *wR* and goodness of fit *S* are based on *F*^2^, conventional *R*-factors *R* are based on *F*, with *F* set to zero for negative *F*^2^. The threshold expression of *F*^2^ > σ(*F*^2^) is used only for calculating *R*-factors(gt) *etc*. and is not relevant to the choice of reflections for refinement. *R*-factors based on *F*^2^ are statistically about twice as large as those based on *F*, and *R*- factors based on ALL data will be even larger.

### Fractional atomic coordinates and isotropic or equivalent isotropic displacement parameters (Å^2^)

**Table:**

	*x*	*y*	*z*	*U*_iso_*/*U*_eq_	Occ. (<1)
Co1	0.5000	0.71666 (2)	0.2500	0.01272 (10)	
Cl1	0.41390 (2)	0.59903 (3)	0.14376 (3)	0.01956 (11)	
N2	0.59089 (8)	0.72168 (10)	0.12398 (11)	0.0151 (3)	
C11	0.65206 (9)	0.79462 (12)	0.14122 (12)	0.0141 (3)	
N1	0.57879 (8)	0.84851 (11)	0.29957 (11)	0.0162 (3)	
C7	0.72069 (9)	0.80426 (13)	0.07300 (13)	0.0171 (3)	
C12	0.64445 (9)	0.86417 (12)	0.23421 (13)	0.0148 (3)	
C3	0.69394 (10)	1.00860 (14)	0.34665 (14)	0.0228 (4)	
H3A	0.7317	1.0626	0.3628	0.027*	
C10	0.59726 (10)	0.65659 (13)	0.03895 (13)	0.0187 (3)	
H10A	0.5558	0.6065	0.0264	0.022*	
C1	0.57135 (10)	0.91093 (14)	0.38597 (14)	0.0215 (4)	
H1A	0.5264	0.9007	0.4311	0.026*	
C6	0.78081 (10)	0.88565 (14)	0.09568 (14)	0.0209 (4)	
H6A	0.8261	0.8924	0.0507	0.025*	
C5	0.77263 (10)	0.95285 (14)	0.18162 (14)	0.0218 (4)	
H5A	0.8120	1.0059	0.1940	0.026*	
C2	0.62797 (10)	0.99184 (15)	0.41280 (15)	0.0253 (4)	
H2A	0.6208	1.0336	0.4748	0.030*	
C8	0.72538 (11)	0.73320 (13)	−0.01602 (14)	0.0210 (4)	
H8A	0.7699	0.7360	−0.0629	0.025*	
C9	0.66376 (10)	0.65987 (14)	−0.03293 (14)	0.0222 (4)	
H9A	0.6660	0.6126	−0.0916	0.027*	
C4	0.70425 (10)	0.94361 (13)	0.25393 (13)	0.0176 (3)	
O1	0.5517 (2)	0.0842 (2)	0.1510 (3)	0.0433 (7)	0.50
N3	0.5000	0.2344 (2)	0.2500	0.0453 (7)	
C13	0.5478 (6)	0.1828 (7)	0.1772 (8)	0.048 (3)	0.50
H13A	0.5840	0.2276	0.1400	0.057*	0.50
C14	0.5000	0.3523 (3)	0.2500	0.0553 (10)	
H14A	0.5551	0.3783	0.2427	0.083*	0.50
H14B	0.4772	0.3783	0.3168	0.083*	0.50
H14C	0.4677	0.3783	0.1904	0.083*	0.50
C15	0.5421 (7)	0.1760 (10)	0.1657 (9)	0.067 (4)	0.50
H15A	0.5375	0.2151	0.0987	0.100*	0.50
H15B	0.5180	0.1055	0.1575	0.100*	0.50
H15C	0.5988	0.1685	0.1846	0.100*	0.50

### Atomic displacement parameters (Å^2^)

**Table:**

	*U*^11^	*U*^22^	*U*^33^	*U*^12^	*U*^13^	*U*^23^
Co1	0.01000 (16)	0.01385 (16)	0.01430 (17)	0.000	−0.00028 (11)	0.000
Cl1	0.0170 (2)	0.0213 (2)	0.0203 (2)	−0.00516 (15)	−0.00274 (15)	−0.00162 (16)
N2	0.0134 (6)	0.0161 (6)	0.0159 (7)	−0.0002 (5)	−0.0015 (5)	0.0012 (5)
C11	0.0124 (7)	0.0146 (7)	0.0152 (8)	0.0011 (6)	−0.0018 (6)	0.0033 (6)
N1	0.0131 (6)	0.0174 (7)	0.0181 (7)	0.0004 (5)	−0.0004 (5)	−0.0016 (6)
C7	0.0148 (8)	0.0187 (8)	0.0177 (8)	0.0012 (6)	0.0003 (6)	0.0036 (6)
C12	0.0130 (7)	0.0147 (7)	0.0168 (8)	0.0012 (6)	−0.0027 (6)	0.0023 (6)
C3	0.0203 (8)	0.0208 (8)	0.0272 (9)	−0.0047 (7)	−0.0045 (7)	−0.0045 (7)
C10	0.0185 (8)	0.0180 (8)	0.0195 (8)	−0.0006 (6)	−0.0012 (7)	−0.0031 (7)
C1	0.0176 (8)	0.0252 (9)	0.0218 (9)	0.0001 (7)	0.0035 (7)	−0.0054 (7)
C6	0.0156 (8)	0.0263 (8)	0.0208 (8)	−0.0033 (7)	0.0022 (7)	0.0047 (7)
C5	0.0181 (8)	0.0236 (8)	0.0238 (9)	−0.0078 (7)	−0.0018 (7)	0.0036 (7)
C2	0.0227 (9)	0.0270 (9)	0.0262 (9)	−0.0020 (7)	0.0003 (7)	−0.0109 (8)
C8	0.0185 (8)	0.0249 (8)	0.0197 (8)	0.0025 (7)	0.0055 (7)	0.0018 (7)
C9	0.0240 (9)	0.0224 (8)	0.0203 (8)	0.0012 (7)	0.0022 (7)	−0.0048 (7)
C4	0.0155 (8)	0.0179 (8)	0.0194 (8)	−0.0003 (7)	−0.0034 (6)	0.0015 (6)
O1	0.0492 (19)	0.0294 (16)	0.0512 (19)	0.0003 (14)	−0.0046 (15)	−0.0048 (14)
N3	0.0390 (15)	0.0223 (12)	0.075 (2)	0.000	−0.0214 (14)	0.000
C13	0.065 (7)	0.018 (4)	0.060 (5)	−0.007 (4)	0.030 (5)	0.001 (3)
C14	0.049 (2)	0.0259 (16)	0.090 (3)	0.000	−0.0091 (19)	0.000
C15	0.047 (5)	0.057 (7)	0.095 (7)	0.001 (5)	−0.041 (5)	−0.028 (5)

### Geometric parameters (Å, °)

**Table:**

Co1—N2	2.1517 (13)	C6—C5	1.353 (2)
Co1—N2^i^	2.1517 (13)	C6—H6A	0.9300
Co1—N1	2.1636 (13)	C5—C4	1.435 (2)
Co1—N1^i^	2.1636 (13)	C5—H5A	0.9300
Co1—Cl1	2.4099 (5)	C2—H2A	0.9300
Co1—Cl1^i^	2.4099 (5)	C8—C9	1.370 (2)
N2—C10	1.326 (2)	C8—H8A	0.9300
N2—C11	1.362 (2)	C9—H9A	0.9300
C11—C7	1.408 (2)	O1—C13	1.260 (9)
C11—C12	1.439 (2)	N3—C13	1.351 (7)
N1—C1	1.321 (2)	N3—C15	1.441 (8)
N1—C12	1.356 (2)	N3—C14	1.456 (4)
C7—C8	1.408 (2)	N3—C13^i^	1.351 (7)
C7—C6	1.433 (2)	N3—C15^i^	1.441 (8)
C12—C4	1.406 (2)	C13—H13A	0.9300
C3—C2	1.368 (2)	C14—H14A	0.9600
C3—C4	1.408 (2)	C14—H14B	0.9600
C3—H3A	0.9300	C14—H14C	0.9600
C10—C9	1.404 (2)	C15—H15A	0.9600
C10—H10A	0.9300	C15—H15B	0.9600
C1—C2	1.401 (2)	C15—H15C	0.9600
C1—H1A	0.9300		
			
N2—Co1—N2^i^	176.70 (7)	C5—C6—C7	121.01 (15)
N2—Co1—N1	76.81 (5)	C5—C6—H6A	119.5
N2^i^—Co1—N1	100.65 (5)	C7—C6—H6A	119.5
N2—Co1—N1^i^	100.65 (5)	C6—C5—C4	121.05 (15)
N2^i^—Co1—N1^i^	76.81 (5)	C6—C5—H5A	119.5
N1—Co1—N1^i^	82.44 (7)	C4—C5—H5A	119.5
N2—Co1—Cl1	91.56 (4)	C3—C2—C1	119.15 (16)
N2^i^—Co1—Cl1	90.43 (4)	C3—C2—H2A	120.4
N1—Co1—Cl1	162.67 (4)	C1—C2—H2A	120.4
N1^i^—Co1—Cl1	87.23 (4)	C9—C8—C7	119.36 (15)
N2—Co1—Cl1^i^	90.43 (4)	C9—C8—H8A	120.3
N2^i^—Co1—Cl1^i^	91.56 (4)	C7—C8—H8A	120.3
N1—Co1—Cl1^i^	87.23 (4)	C8—C9—C10	119.48 (15)
N1^i^—Co1—Cl1^i^	162.67 (4)	C8—C9—H9A	120.3
Cl1—Co1—Cl1^i^	105.91 (2)	C10—C9—H9A	120.3
C10—N2—C11	117.80 (14)	C12—C4—C3	117.06 (15)
C10—N2—Co1	127.52 (11)	C12—C4—C5	119.30 (15)
C11—N2—Co1	114.39 (10)	C3—C4—C5	123.62 (15)
N2—C11—C7	123.19 (14)	C13^i^—N3—C13	123.8 (8)
N2—C11—C12	117.10 (14)	C13—N3—C15^i^	121.4 (4)
C7—C11—C12	119.71 (14)	C13^i^—N3—C15	121.4 (4)
C1—N1—C12	118.02 (14)	C15^i^—N3—C15	120.0 (11)
C1—N1—Co1	127.74 (11)	C13—N3—C14	118.1 (4)
C12—N1—Co1	114.20 (10)	C15—N3—C14	120.0 (6)
C11—C7—C8	117.23 (15)	O1—C13—N3	130.9 (6)
C11—C7—C6	119.27 (15)	O1—C13—H13A	114.6
C8—C7—C6	123.49 (15)	N3—C13—H13A	114.6
N1—C12—C4	123.13 (14)	N3—C14—H14A	109.5
N1—C12—C11	117.25 (14)	N3—C14—H14B	109.5
C4—C12—C11	119.62 (14)	H14A—C14—H14B	109.5
C2—C3—C4	119.56 (16)	N3—C14—H14C	109.5
C2—C3—H3A	120.2	H14A—C14—H14C	109.5
C4—C3—H3A	120.2	H14B—C14—H14C	109.5
N2—C10—C9	122.94 (15)	N3—C15—H15A	109.5
N2—C10—H10A	118.5	N3—C15—H15B	109.5
C9—C10—H10A	118.5	H15A—C15—H15B	109.5
N1—C1—C2	123.06 (16)	N3—C15—H15C	109.5
N1—C1—H1A	118.5	H15A—C15—H15C	109.5
C2—C1—H1A	118.5	H15B—C15—H15C	109.5
			
N1—Co1—N2—C10	178.15 (14)	N2—C11—C12—N1	2.6 (2)
N1^i^—Co1—N2—C10	−102.31 (13)	C7—C11—C12—N1	−177.44 (14)
Cl1—Co1—N2—C10	−14.83 (13)	N2—C11—C12—C4	−177.75 (14)
Cl1^i^—Co1—N2—C10	91.10 (13)	C7—C11—C12—C4	2.2 (2)
N1—Co1—N2—C11	4.54 (10)	C11—N2—C10—C9	0.1 (2)
N1^i^—Co1—N2—C11	84.08 (11)	Co1—N2—C10—C9	−173.37 (12)
Cl1—Co1—N2—C11	171.56 (10)	C12—N1—C1—C2	−0.1 (2)
Cl1^i^—Co1—N2—C11	−82.51 (10)	Co1—N1—C1—C2	177.45 (13)
C10—N2—C11—C7	0.5 (2)	C11—C7—C6—C5	0.2 (2)
Co1—N2—C11—C7	174.73 (12)	C8—C7—C6—C5	179.06 (16)
C10—N2—C11—C12	−179.63 (14)	C7—C6—C5—C4	1.1 (3)
Co1—N2—C11—C12	−5.35 (17)	C4—C3—C2—C1	−0.8 (3)
N2—Co1—N1—C1	179.24 (15)	N1—C1—C2—C3	0.6 (3)
N2^i^—Co1—N1—C1	1.40 (15)	C11—C7—C8—C9	0.7 (2)
N1^i^—Co1—N1—C1	76.37 (14)	C6—C7—C8—C9	−178.19 (16)
Cl1—Co1—N1—C1	130.31 (14)	C7—C8—C9—C10	−0.2 (2)
Cl1^i^—Co1—N1—C1	−89.67 (14)	N2—C10—C9—C8	−0.2 (3)
N2—Co1—N1—C12	−3.15 (10)	N1—C12—C4—C3	0.1 (2)
N2^i^—Co1—N1—C12	179.00 (10)	C11—C12—C4—C3	−179.44 (14)
N1^i^—Co1—N1—C12	−106.02 (12)	N1—C12—C4—C5	178.67 (15)
Cl1—Co1—N1—C12	−52.08 (19)	C11—C12—C4—C5	−0.9 (2)
Cl1^i^—Co1—N1—C12	87.94 (10)	C2—C3—C4—C12	0.4 (2)
N2—C11—C7—C8	−0.8 (2)	C2—C3—C4—C5	−178.04 (17)
C12—C11—C7—C8	179.25 (14)	C6—C5—C4—C12	−0.7 (2)
N2—C11—C7—C6	178.10 (14)	C6—C5—C4—C3	177.71 (16)
C12—C11—C7—C6	−1.8 (2)	C13^i^—N3—C13—O1	9.9 (10)
C1—N1—C12—C4	−0.3 (2)	C15^i^—N3—C13—O1	18 (2)
Co1—N1—C12—C4	−178.17 (12)	C15—N3—C13—O1	−62 (7)
C1—N1—C12—C11	179.28 (14)	C14—N3—C13—O1	−170.1 (10)
Co1—N1—C12—C11	1.42 (17)		

Symmetry codes: (i) −*x*+1, *y*, −*z*+1/2.

### Hydrogen-bond geometry (Å, °)

**Table:**

*D*—H···*A*	*D*—H	H···*A*	*D*···*A*	*D*—H···*A*
C10—H10A···Cl1	0.93	2.74	3.3408 (17)	124.
C6—H6A···Cl1^ii^	0.93	2.80	3.6743 (18)	158.
C5—H5A···Cl1^iii^	0.93	2.85	3.6375 (17)	144.
C2—H2A···Cg1^iv^	0.93	2.99	3.768 (2)	142
C8—H8A···Cg2^v^	0.93	2.90	3.608 (2)	134

Symmetry codes:  (ii) *x*+1/2, −*y*+3/2, −*z*; (iii) *x*+1/2, *y*+1/2, −*z*+1/2; (iv) *x*, −*y*+1, *z*−1/2; (v) −*x*+3/2, −*y*+1/2, *z*−1/2.
